# Corneal collagen cross-linking in a late-onset graft infectious ulcer: a case report

**DOI:** 10.1186/1752-1947-8-180

**Published:** 2014-06-06

**Authors:** Georgios Labiris, Athanassios Giarmoukakis, Roman Larin, Haris Sideroudi, Vassilios P Kozobolis

**Affiliations:** 1 Department of Ophthalmology, University Hospital of Alexandroupolis, 68100 Dragana, Alexandroupolis, Greece; 2 Eye Institute of Thrace, Alexandroupolis, Alexandroupolis, Greece

**Keywords:** Corneal collagen cross-linking, Corneal ulcer, Penetrating keratoplasty

## Abstract

**Introduction:**

Infectious keratitis following penetrating keratoplasty is a common postoperative complication. Intensive topical and systemic treatments do not always prevent the risk of graft failure. In this report we demonstrate the beneficial anti-microbial effect of corneal collagen cross-linking in a late-onset, sight-threatening, corneal graft ulcer.

**Case presentation:**

A 57-year old Caucasian man underwent penetrating keratoplasty in his left eye, due to corneal bullosa after cataract extraction surgery. Twelve months after the penetrating keratoplasty, he visited our emergency service complaining of burning and foreign body sensation in his operated eye. Slit-lamp examination revealed a central, round-shaped ulcer of the graft. Due to poor response to the intensive topical antibiotic therapy, corneal collagen cross-linking was applied 3 days after admission, in an attempt to control the infection. Cultures indicated the predominance of methicillin-resistant *Staphylococcus aureus* infection. Five days after corneal collagen cross-linking treatment, the epithelium was completely re-epithelized, while the transparency of the transplanted cornea was gradually restored within the 12-month follow-up period. No relapses occurred.

**Conclusion:**

Corneal collagen cross-linking seems to be a safe and effective therapeutic alternative in resistant cases of infectious keratitis following penetrating keratoplasty.

## Introduction

Infectious keratitis after penetrating keratoplasty (PK) is a common, sight-threatening complication both in the early and the late postoperative period. Post-PK infectious keratitis is associated with a dramatic decrease in visual acuity and graft decompensation [1]. Unfortunately, intensive topical and systemic medications do not always prevent graft failure and/or permanent visual impairment [1].

Corneal collagen cross-linking (CxL) is a minimally invasive technique that combines the use of riboflavin and ultraviolet A (UV-A) radiation, enhancing the rigidity of the corneal tissue [2]. Despite the fact that the primary indication for CxL is the management of ectatic disorders [3], recent publications indicate that it might be used as a therapeutic alternative in a series of other corneal diseases like infectious keratitis and corneal bullosa [4-9].

In this report, we demonstrate the successful therapeutic management of a resistant infectious keratitis following penetrting keratoplasty by means of CxL.

## Case presentation

We describe the case of a 57-year-old Caucasian man with uneventful medical history who underwent complicated phacoemulsification cataract surgery (posterior capsular rupture with anterior vitrectomy) and intraocular lens insertion at the sulcus of his left eye, in a private medical center. He was referred to our department 1 year after his initial cataract operation due to endothelial insufficiency and elevated intraocular pressure (IOP) in his operated eye. At presentation he presented best corrected visual acuity (BCVA) of counting fingers (CF) at 1 meter and, IOP of 28mmHg despite intensive topical treatment of timolol–dorzolamide fixed combination (Cosopt®, Vianex AE, Greece) twice a day, brimonidine (Alphagan®, Allergan Pharmaceuticals Ltd, Ireland) twice a day and latanoprost (Xalatan®, Pfizer Hellas A.E., Greece) once per day. Slit-lamp biomicroscopy revealed a typical picture of corneal bullosa. His right eye presented BCVA 20/20 and IOP of 14mmHg without any medication.

He was admitted and underwent uneventful PK in his left eye, followed by an uneventful trabeculectomy with mitomycin, 3 months later. Within 6 months after the PK his diurnal IOP measurements had been stabilized (mean IOP: 15mmHg) without any glaucoma medication, while his BCVA remained CF at 1 meter due to non-reversible damage to his optic nerve by the uncontrolled IOP in the period following the cataract operation. His corneal graft was clear with no signs of rejection, and he was tapering loteprednol (Lotemax®, Kite Hellas EPE, Greece) drops.

**Figure 1:**
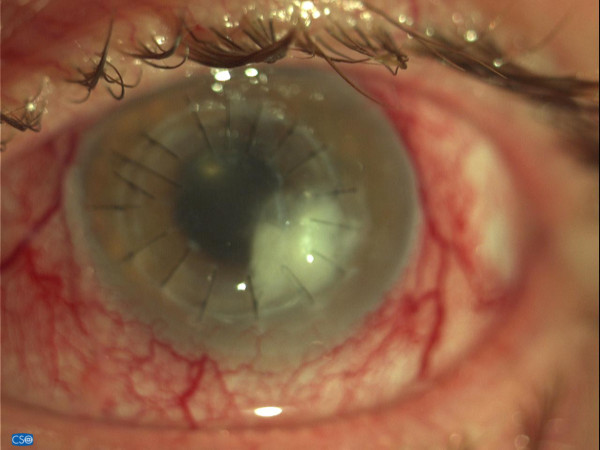
Graft ulcer at presentation.

**Figure 2:**
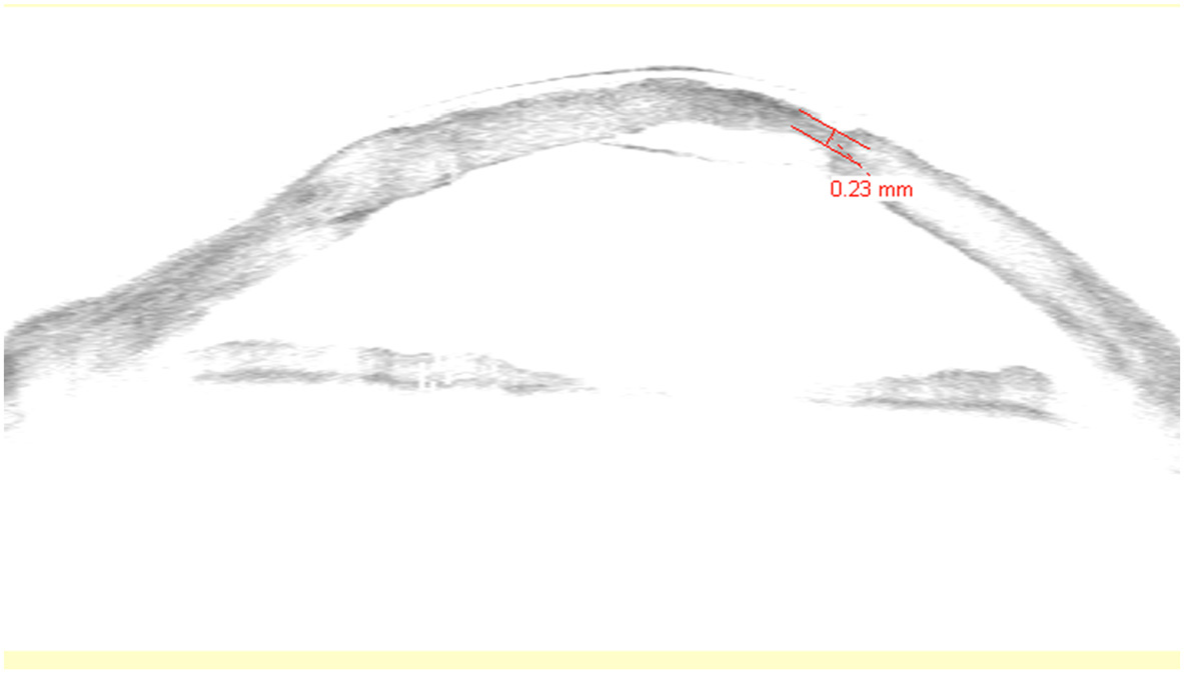
Anterior segment optical coherence tomography of the ulcer.

Twelve months following the PK and prior to any suture removal, he urgently visited our emergency service, complaining of burning and foreign body sensation in the operated eye. His visual acuity had decreased to hand motion and slit-lamp biomicroscopy revealed conjunctival injection and a central, round-shaped ulcer of the graft, extending from 3 to 5 o’-clock area, with a size of 3 to 3.5mm (Figure 1). Moreover, anterior-segment optical coherence tomography demonstrated severe thinning of the graft at the site of the ulcer with associated local Descemet-membrane detachment (Figure 2). Gram and Giemsa stains, as well as cultures of the affected corneal tissue were performed, while two sutures at the site of the ulcer, and two more at the contralateral site, were removed. The sight-threatening clinical picture and the positive results for cocci from the stains suggested the introduction of intensive topical antibiotic therapy that included fortified 5% vancomycin (Voncon®, Lilly, Nederland B.V.) alternating with fortified 5% ceftazidime (Solvetan®, GlaxoSmithKline ABEE, Greece), every 30 minutes. However, despite the intensive antibiotic treatment, the clinical picture continued to deteriorate with visualization of inflammatory cells in the anterior chamber and destabilization of the graft-recipient cornea connection at the site of the ulcer. Meanwhile, the stains indicated a multimicrobial infection and the cultures isolated methicillin-resistant *Staphylococcus aureus* (MRSA). Based on the clinical and laboratory data, CxL was attempted 3 days after the emergency admission in order to control the infection and stabilize the border between the graft and the recipient cornea.

**Figure 3:**
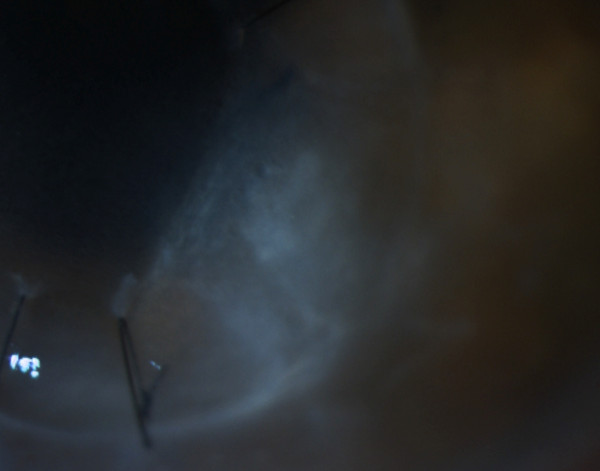
Imaging of the affected area (5th day post-collagen cross-linking).

**Figure 4:**
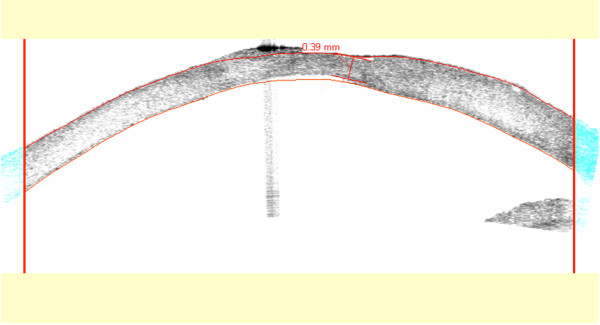
Anterior segment optical coherence tomography 1 month post-collagen cross-linking.

CxL treatment was applied as follows: proparacaine hydrochloride 0.5% drops were instilled for topical anesthesia. De-epithelialization was performed by means of a hockey knife, in a circular area that surrounded the ulcer and at least 2mm of healthy corneal tissue. After de-epithelialization, a mixture of 0.1% riboflavin in 20% dextran solution was instilled to his cornea (two drops every 2 minutes) until the stroma was completely penetrated and the aqueous was stained yellow. In our case we could visualize the change in the color of the aqueous about 14 minutes after the instillation had begun. The UV-A radiation source that was used was the UV-X (Peschke Meditrade, Cham, Switzerland). In detail, an 8.0mm diameter of central cornea was irradiated for 30 minutes by UV-A light with a wavelength of 370nm and an irradiance of 3mW/cm^2^, while instillation of riboflavin drops (one drop every 2 minutes) was continued during irradiation. Moreover, a balanced salt solution was applied every 6 minutes to moisten his cornea. After irradiation, a soft contact lens (Day & Night; CIBA Vision, Corp, Duluth, GA, USA) was applied.Within 5 days after the CxL, significant improvement of the clinical picture was observed (Figure 3), the epithelium was completely re-epithelized, and the topical treatment was modified to moxifloxacin (Vigamox®, Alcon Laboratories AEBE, Greece) drops, four times a day. The topical antibiotic treatment was continued for 1 month following CxL treatment. The final postoperative examination revealed minor scarring at the site of the ulcer with strong connection between the graft and recipient cornea (Figure 4). No relapses occurred for a total postoperative period of 12 months.

## Discussion

It is well established that the activation of riboflavin, during the CxL process, is accompanied by release of reactive oxygen species. These oxygen species combined with the antimicrobial activity of UV-A radiation itself can induce deoxyribonucleic acid (DNA) damage to a series of pathogens [10]. Martins *et al*. [5] demonstrated the antimicrobial properties of CxL against common pathogens. Moreover, it is already known that post-CxL corneas demonstrate increased tissue resistance to microbial enzymatic digestion [2].

An extensive literature review revealed a series of reports on successful CxL application in cases of infectious keratitis. Iseli *et al*. [6] described several cases of bacterial keratitis, in which CxL treatment inhibited the corneal melting process. In their study, Kozobolis *et al*. [4] presented a case series of bullous keratopathy combined with corneal ulcer that was successfully treated with CxL. In our case, the infectious ulcer was developed on a corneal transplant. The clinical examination and the Gram stains suggested a multimicrobial infection, while the cultures isolated MRSA. It is known that Staphylococcus aureus is among the most common causes of late microbial keratitis, following PK [11,12], mainly due to the long-term use of topical steroids to prevent rejection [13,14]. Therefore, intensive topical antibiotic therapy, including fortified 5% vancomycin drops, was initiated upon admission. Despite that fact, the clinical picture continued to deteriorate for the following 2 days. Therefore, CxL treatment was applied, in an attempt to: a) control the infection, b) prevent corneal perforation, and c) prevent the destabilization of the graft-recipient corneal bond. Since no guidelines have been introduced for CxL in infectious keratitis, we modified the standard protocol prior to UV-A irradiation, as follows: a) de-epithelialization was attempted only around the affected area, and b) we continued UV-A irradiation immediately when aqueous was stained yellow. However, a full 30 minutes of UV-A irradiation was applied. The outcome of CxL treatment was at least encouraging; the deteriorating clinical picture was completely reversed, infection was controlled and the graft survived. The authors cannot predict whether the conventional topical antibiotic treatment alone would produce the same favorable outcome, however, we are confident that CxL had a beneficial synergetic effect without any evident negative impact on the graft.

## Conclusions

To the best of our knowledge, this is the first case of successful application of CxL for the treatment of infectious keratitis on a corneal graft. The results of our report are encouraging, regarding the potential therapeutic application of CxL in patients with sight-threatening, post-PK infectious keratitis. Further studies are necessary to explore the potential beneficial impact of the proposed therapeutic procedure and provide the necessary data for the development of a valid therapeutic protocol.

## Consent

Written informed consent was obtained from the patient for publication of this case report and accompanying images. A copy of the written consent is available for review by the Editor-in-Chief of this journal.

## Abbreviations

BCVA: Best corrected visual acuity; CF: Counting fingers; CxL: Corneal collagen cross-linking; IOP: Intraocular pressure; MRSA: Methillicin-resistant Staphylococcus aureus; UV-A: Ultraviolet A.

## Competing interests

The authors declare that they have no competing interests.

## Authors’ contributions

GL provided the diagnostic/therapeutic management of the patient and wrote the writing of the manuscript. AG was involved in draft writing. RL assisted in the therapeutic management. HS revised the manuscript. VK coordinated the project. All authors read and approved the final manuscript.
